# Sodium–Glucose Cotransporter 2-Mediated Glucose Uptake Contributes to Glycogen Accumulation in Clear Cell Renal Cell Carcinoma: Implications for Clear Cell Morphology and Potential Therapeutic Relevance

**DOI:** 10.3390/ijms27209128

**Published:** 2026-10-15

**Authors:** Yujiro Nagata, Ikko Tomisaki, Takuo Matsukawa, Nguyen Thu Quynh, Yuto Tsubonuma, Yoshihiro Sugita, Aya Nawata, Keiji Kokubu, Ryoko Baba, Hiroyuki Morimoto, Kohei Ogawa, Koichiro Wada, Naohiro Fujimoto

**Affiliations:** 1 Department of Urology, School of Medicine, University of Occupational and Environmental Health, Kitakyushu 807-8555, Fukuoka, Japan; ikko@med.uoeh-u.ac.jp (I.T.); takuomatsukawa@med.uoeh-u.ac.jp (T.M.); quynh@med.uoeh-u.ac.jp (N.T.Q.); yuto09220304@gmail.com (Y.T.); sugiyoshi@med.uoeh-u.ac.jp (Y.S.); n-fuji@kurate-hp.com (N.F.); 2 Department of Urology, Shimane University Faculty of Medicine, Izumo 693-8501, Shimane, Japan; k.ogawa@med.shimane-u.ac.jp (K.O.); wada-uro@med.shimane-u.ac.jp (K.W.); 3 Department of Pathology and Oncology, School of Medicine, University of Occupational and Environmental Health, Kitakyushu 807-8555, Fukuoka, Japan; aya.y0116@gmail.com; 4 Department of Anatomy, School of Medicine, University of Occupational and Environmental Health, Kitakyushu 807-8555, Fukuoka, Japan; kokubu@med.uoeh-u.ac.jp (K.K.); babar@med.uoeh-u.ac.jp (R.B.); morimoto@med.uoeh-u.ac.jp (H.M.); 5 Department of Urology, Kurate Hospital, Kurate 807-1311, Fukuoka, Japan

**Keywords:** clear cell renal cell carcinoma, molecular pathology, morphology, SGLT2, glycogen, glucose uptake, VHL

## Abstract

Clear cell renal cell carcinoma (ccRCC) is characterized by loss of the functional von Hippel–Lindau (VHL) complex and distinctive clear cell morphology associated with intracellular glycogen and lipid accumulation. However, the mechanism underlying glycogen storage remains unclear. This study was conducted to investigate the relationship between SGLT2, a glucose transporter predominantly expressed in proximal tubular epithelial cells; the cell of origin of ccRCC; and clear cell morphology in ccRCC. Using the VHL-null ccRCC cell line KMRC-1, we investigated the effects of the SGLT2 inhibitor dapagliflozin, SGLT2 knockdown, and VHL overexpression on cellular morphology, intracellular glycogen accumulation, and glucose uptake. Immunohistochemical expression of SGLT2, glucose transporter 1, and VHL was evaluated in morphologically heterogeneous ccRCC specimens. Periodic acid–Schiff (PAS) and PAS with diastase staining, together with quantitative image analysis of PAS-stained cells, showed that dapagliflozin treatment, SGLT2 knockdown, and VHL restoration reduced cytoplasmic area and cytoplasmic PAS staining intensity in KMRC-1 cells. In the fluorescent glucose uptake assay, dapagliflozin significantly reduced the fluorescence signal (*p* < 0.05). In paired intratumoral analyses of six VHL-deficient ccRCC specimens, SGLT2 immunohistochemical scores were significantly higher in clear cell components than in eosinophilic components (*p* = 0.026), whereas GLUT1 scores did not differ significantly (*p* = 0.850). These findings suggest that SGLT2-mediated glucose uptake contributes to glycogen accumulation and the glycogen-rich clear cell morphology of VHL-deficient ccRCC. The potential therapeutic relevance of SGLT2 inhibition in ccRCC warrants further investigation.

## 1. Introduction

Renal cell carcinoma (RCC) constitutes the majority of renal malignancies [1,2]. Among its histological subtypes, clear cell RCC (ccRCC) is the most common [2]. ccRCC originates from proximal tubular epithelial cells (PTECs) [3,4] and is characterized histologically by the cytoplasmic accumulation of glycogen and lipids [5,6] that are removed in standard histological preparations [7]. Notably, this histological subtype is rare among primary malignancies of the other organs.

There have been extensive investigations into the etiology of ccRCC. For instance, large-scale genomic analyses of sporadic ccRCC conducted by The Cancer Genome Atlas revealed that functional loss of the von Hippel–Lindau (VHL) complex occurs in 90% of cases due to mutations in the *VHL* tumor suppressor gene, DNA methylation of the *VHL* promoter, or mutations in *TCEB1*, a component of the VHL complex [8,9]. Moreover, alterations in *VHL* act as driver mutations contributing directly to the development of ccRCC. Loss of function of the VHL complex inhibits the degradation of hypoxia-inducible factor (HIF)-1α and HIF-2α, resulting in metabolic reprogramming mimicking a hypoxic environment [10,11,12]. This metabolic adaptation requires an increase in glucose uptake, partially through the upregulation of glucose transporter 1 (GLUT1) to sustain energy production via anaerobic respiration [10,11,12,13,14]. Remarkably, RCC cell lines with mutations in *VHL* exhibit greater quantities of carbohydrates than those with wild-type *VHL* [15]. Interestingly, activation of HIF-2α causes abnormal glycogen accumulation, even in benign PTECs [16].

Patients with VHL disease, characterized by autosomal dominant *VHL* mutations, develop ccRCC at high frequencies (24–45%), which further implicates *VHL* abnormalities in ccRCC oncogenesis [17]. In particular, although VHL disease predisposes patients to various benign and malignant tumors, only RCC exhibits the characteristic clear cell phenotype associated with abundant intracellular glycogen accumulation, suggesting that loss of the functional VHL complex alone is insufficient to generate the clear cell phenotype of ccRCC.

A functional characteristic of PTECs, the cells from which ccRCC originates, is glucose reabsorption from the glomerular filtrate [18]. Under normoglycemic conditions, approximately 180 g/day of glucose is filtered into the glomerular filtrate, with almost the entire amount being reabsorbed by four glucose transporters in the PTECs. Among these transporters, sodium–glucose cotransporter (SGLT)2, which is encoded by the *SLC5A2* gene and predominantly expressed in PTECs, is responsible for most of this filtered glucose reabsorption [18]. In normal PTECs, glucose transport is segmentally organized. SGLT2 is localized to the early S1 segment, where it reabsorbs approximately 80–90% of filtered glucose from the tubular lumen, with glucose subsequently transported across the basolateral membrane, primarily via GLUT2. In the more distal S2/S3 segments, SGLT1 reabsorbs the remaining 10–20% of filtered glucose, whereas GLUT1 mediates basolateral glucose transport into the interstitial space [18]. Thus, although SGLT2 and GLUT1 both participate in glucose handling in PTECs, they differ in their tubular distribution, membrane localization, and transport mechanisms. SGLT2 mediates sodium-dependent glucose cotransport, whereas GLUT1 facilitates sodium-independent glucose transport. Not surprisingly, ccRCC cells continue to express SGLT2 [19,20]. Moreover, ccRCC cells overexpress glycogen synthase 1, suggesting that large amounts of glucose taken up by ccRCC cells are partially used for glycogen synthesis [21,22]. In ccRCC, both transporters are expressed [19], suggesting that tumor cells may utilize multiple glucose uptake pathways distinct from the physiological segmental organization of normal PTECs. On the basis of these findings, we hypothesize that the “clear cell” characteristic of ccRCC is inherently linked to the PTEC lineage following loss of VHL function and that ccRCC cells actively take up large quantities of glucose via SGLT2, which is subsequently used for glycogen synthesis and accumulation.

Although these observations collectively suggest the relevance of SGLT2 in ccRCC, there is limited information on SGLT2 expression in ccRCC and its biological implications. To date, there have been no studies on the relationship between SGLT2 and the morphological characteristics of ccRCC. Therefore, the aim of this study was to determine whether SGLT2-mediated glucose uptake contributes to glycogen accumulation and the clear cell morphology of ccRCC.

## 2. Results

### 2.1. Expression of VHL in Human ccRCC Cells

**Figure 1:**
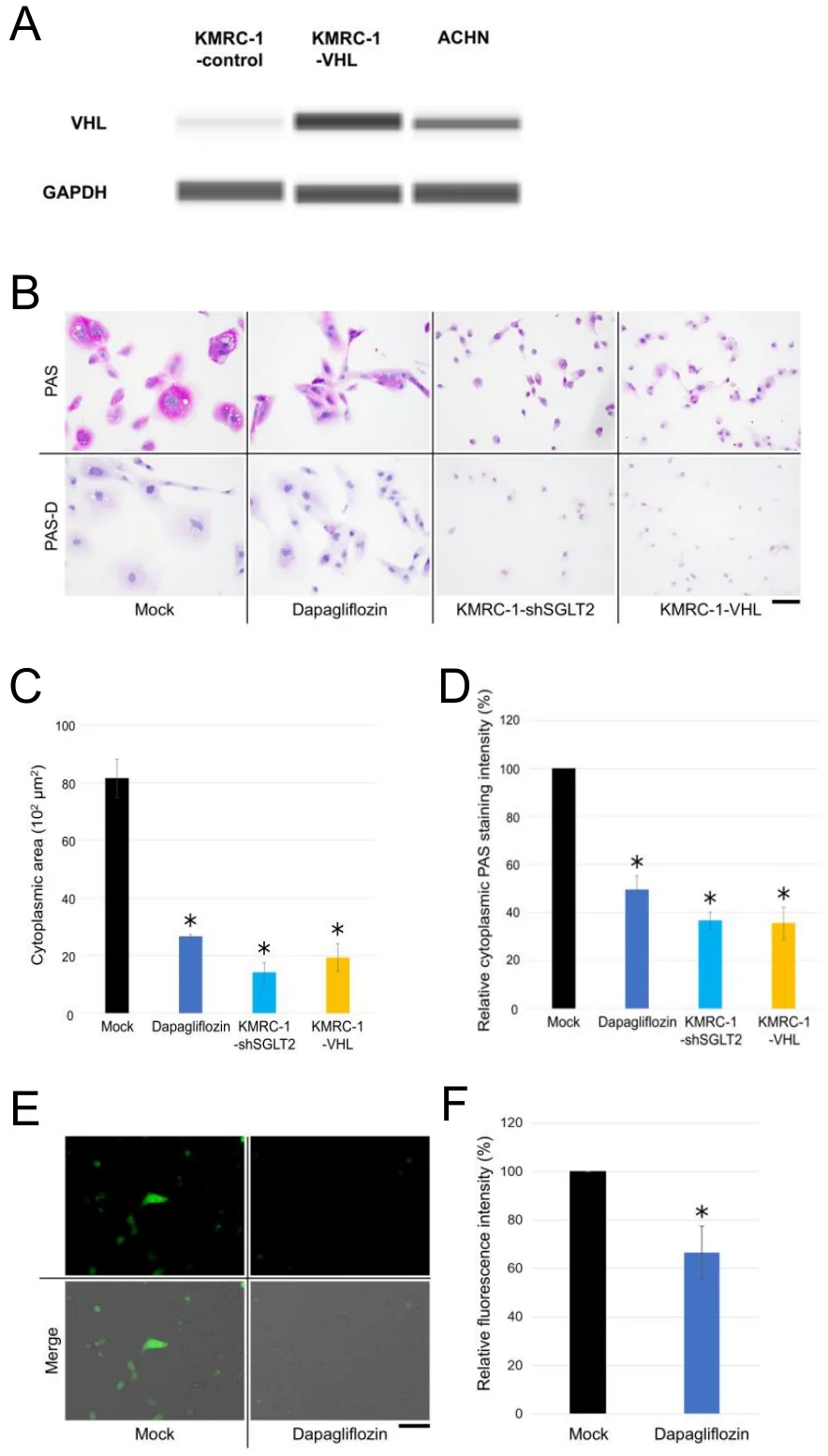
Effects of SGLT2 inhibition and VHL restoration on glucose uptake and associated glycogen accumulation in VHL-deficient ccRCC cells. (**A**) Expression of VHL protein in sublines of the VHL-deficient ccRCC cell lines KMRC-1 and ACHN (*VHL* wild-type), as determined by Western blotting using the Simple Western™ system. Stable overexpression of VHL was confirmed in KMRC-1-VHL cells. GAPDH served as a loading control. Images are representative of three independent experiments. (**B**) Periodic acid–Schiff (PAS) and PAS with diastase (PAS-D) staining of KMRC-1 cells and their sublines. Pronounced cytoplasmic glycogen accumulation was observed in KMRC-1 cells (mock treatment), whereas cytoplasmic area and PAS-positive staining were markedly reduced following SGLT2 inhibition (dapagliflozin), SGLT2 knockdown, and VHL restoration. Magnification ×200. Scale bar, 100 µm. (**C**) Quantitative analysis of cytoplasmic area in PAS-stained mock-treated, dapagliflozin-treated, SGLT2-knockdown, and VHL-restored KMRC-1 cells. (**D**) Quantitative analysis of mean cytoplasmic PAS staining intensity in the same experimental groups. PAS staining intensity was normalized to the corresponding mock control in each independent experiment and expressed as a percentage. For (**C**,**D**), three independent staining experiments were analyzed, with 15 cells evaluated per condition in each experiment. Measurements from the 15 cells were averaged within each experiment, and the experiment-level means were used as biological replicates (*n* = 3). Data are presented as the mean ± SD. Statistical significance was assessed using one-way repeated-measures ANOVA followed by Bonferroni-adjusted pairwise comparisons. (**E**) Representative fluorescence microscopy images showing intracellular glucose uptake in KMRC-1 cells after treatment with the SGLT2 inhibitor dapagliflozin (2 μM, 96 h), using a green fluorescent glucose analog. Cells with abundant cytoplasm exhibited prominent glucose uptake under control conditions. Magnification ×200. Scale bar, 100 µm. (**F**) Quantification of intracellular glucose uptake shown in (**E**). Fluorescence intensity is expressed relative to mock-treated cells and presented as mean ± SD of three independent experiments. * *p* < 0.05 vs. mock treatment.

Using an SGLT2-positive, VHL-negative KMRC-1 ccRCC line [20], we confirmed successful VHL overexpression, as demonstrated by a considerable increase in VHL protein levels in the overexpression subline (KMRC-1-VHL) detected by Western blotting (Figure 1A). An RCC cell line with wild-type *VHL* (ACHN) served as a positive control for VHL [23].

### 2.2. Glycogen-Associated PAS Staining and Quantitative Image Analysis

Cytoplasmic glycogen-associated staining was evaluated via periodic acid–Schiff (PAS) staining, with diastase digestion (PAS-D) used as a histochemical specificity control in ccRCC and its sublines (Figure 1B). KMRC-1 cells demonstrated abundant PAS-positive cytoplasm, consistent with our previous report [15]. In contrast, dapagliflozin-treated KMRC-1, KMRC-1-shSGLT2, and KMRC-1-VHL cells showed marked reductions in both cytoplasmic area and PAS-positive staining.

To quantitatively assess these changes, cytoplasmic area and mean cytoplasmic PAS staining intensity were measured in three independent experiments (Figure 1C,D). Cytoplasmic area was significantly reduced in the dapagliflozin-treated, SGLT2-knockdown, and VHL-restored cells compared with the mock treatment cells (mock, 8150.2 ± 668.7 µm^2^; dapagliflozin, 2660.1 ± 68.0 µm^2^; SGLT2 knockdown, 1412.1 ± 352.7 µm^2^; VHL restoration, 1927.7 ± 488.0 µm^2^; mean ± SD, *n* = 3). The overall difference among conditions was significant by one-way repeated-measures ANOVA (*p* < 0.001), and Bonferroni-adjusted pairwise comparisons showed significant reductions versus mock for dapagliflozin (*p* = 0.035), SGLT2 knockdown (*p* = 0.009), and VHL restoration (*p* = 0.037) (Figure 1C).

Mean cytoplasmic PAS staining intensity was also significantly reduced under all three conditions (raw intensity: mock, 114.3 ± 3.2 a.u.; dapagliflozin, 56.5 ± 5.2 a.u.; SGLT2 knockdown, 41.9 ± 3.6 a.u.; VHL restoration, 40.7 ± 8.4 a.u.; mean ± SD, *n* = 3). The overall difference among conditions was significant (*p* < 0.001). Bonferroni-adjusted pairwise comparisons showed reductions for dapagliflozin (*p* = 0.038), SGLT2 knockdown (*p* = 0.010), and VHL restoration (*p* = 0.015) verses mock (Figure 1D). These findings indicate that the reduction in PAS staining was not attributable solely to the decrease in cytoplasmic area, because mean PAS staining intensity within the cytoplasmic region was also reduced.

### 2.3. Glucose Uptake

Intracellular glucose uptake was determined using a fluorescent glucose analog (Figure 1E,F), which showed that the fluorescence signal in the glucose uptake assay was significantly reduced by dapagliflozin treatment in KMRC-1 cells. In particular, probe uptake was prominent in cells with abundant cytoplasm (Figure 1E).

### 2.4. Microscopic and Immunohistochemical Intratumoral Heterogeneity Within Primary Tumors

**Figure 2:**
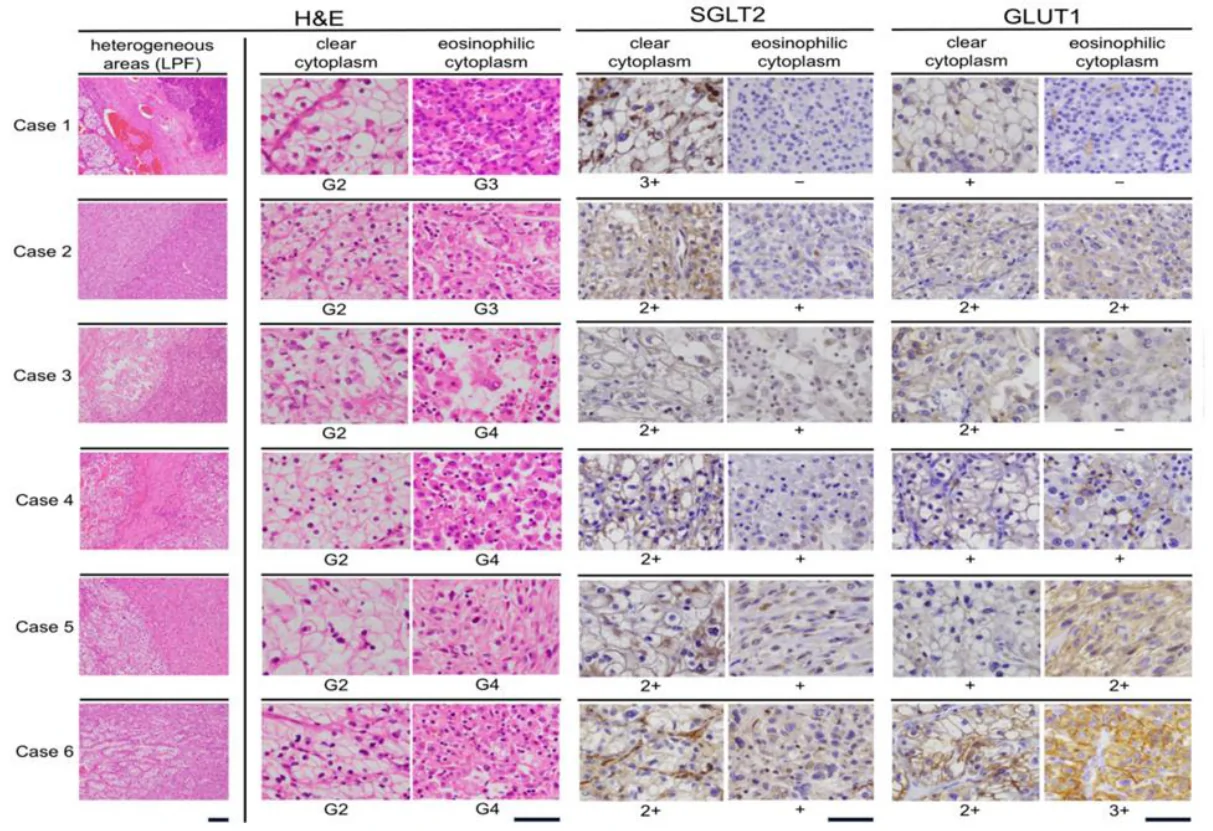
Intratumoral heterogeneity in primary ccRCC specimens and corresponding SGLT2/GLUT1 expression. Representative hematoxylin and eosin (H&E) staining images of six primary ccRCC specimens showing adjacent typical clear cell (low-grade) and eosinophilic (high-grade) components within the same tumor. SGLT2 and GLUT1 staining intensities were semiquantitatively scored as 0 (negative), 1+ (weak), 2+ (moderate), and 3+ (strong). SGLT2 staining scores were significantly higher in clear cell components than in paired eosinophilic components (*p* = 0.026, Wilcoxon signed-rank test), whereas GLUT1 scores did not differ significantly (*p* = 0.850). G, WHO/ISUP grade; LPF, low power field. Scale bar, 200 µm (LPF) and 50 µm (others).

**Figure 3:**
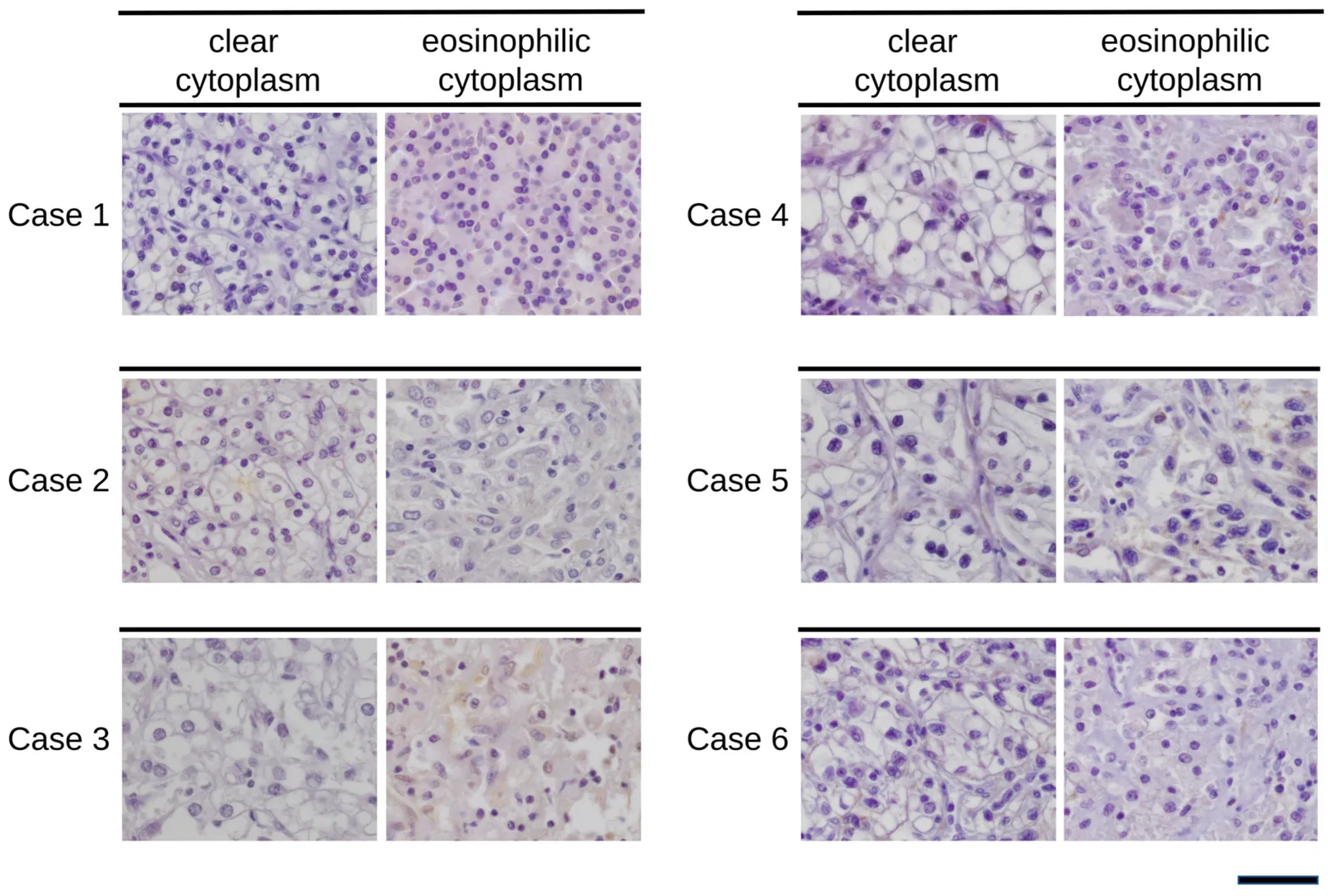
Absent VHL immunoreactivity in primary ccRCC specimens. Representative immunohistochemical staining for VHL in six primary ccRCC specimens shows absent tumor cell staining. Scale bar, 50 µm.

We examined specimens obtained from six patients with sporadic ccRCC exhibiting different phenotypes within spatially distinct regions of adjacent clear cell and eosinophilic tumors, all of whom had undergone radical nephrectomy. Table 1 summarizes the baseline characteristics of these patients. All the specimens demonstrated intratumoral heterogeneity, exhibiting adjacent typical clear cell and eosinophilic tumors under microscopic examination. Hematoxylin and eosin staining revealed low-grade clear cell tumors with adjacent high-grade eosinophilic tumors in ccRCC (Figure 2). We examined the immunohistochemical expression levels of SGLT2, GLUT1, and VHL (Figure 2), finding that SGLT2’s immunohistochemical scores were higher in the clear cell component than in the paired eosinophilic component in all six cases, with a statistically significant difference between the two components (*p* = 0.026, Wilcoxon signed-rank test). In contrast, GLUT1 expression showed no consistent difference between the two morphological components, and the paired immunohistochemical scores did not differ significantly (*p* = 0.850, Wilcoxon signed-rank test). VHL expression was negative in all six cases (Figure 3).

### 2.5. Impact of SGLT2 Under GLUT1 Inactivation on ccRCC Cell Viability

**Figure 4:**
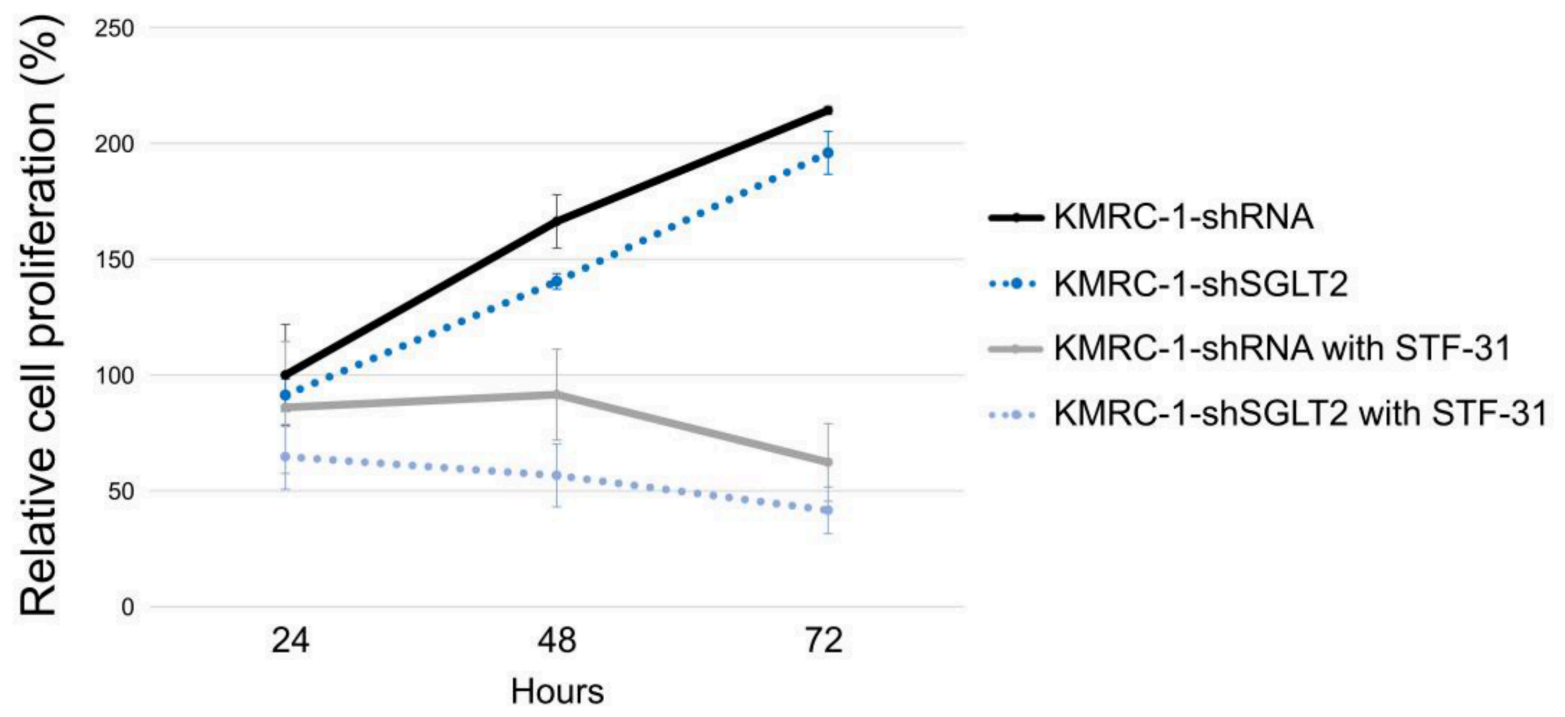
Effect of GLUT1 inhibition on the viability of SGLT2-knockdown ccRCC cells. MTT assay of KMRC-1-shRNA (control) and KMRC-1-shSGLT2 (SGLT2 knockdown) sublines treated with dimethyl sulfoxide (DMSO; mock) or the GLUT1 inhibitor STF-31 (10 µM). Cell viability is represented as the mean ± SD of three independent experiments. Statistical analysis was performed separately at each time point using two-way ANOVA with SGLT2 knockdown and STF-31 treatment as the two factors, including their interaction. Significant main effects of both SGLT2 knockdown and STF-31 treatment were observed at 48 h (*p* = 0.002 and *p* < 0.001, respectively) and 72 h (*p* = 0.004 and *p* < 0.001, respectively). No significant interaction between SGLT2 knockdown and STF-31 treatment was observed at 24, 48, or 72 h (*p* = 0.537, 0.520, and 0.879, respectively).

We compared the effect of GLUT1 inhibition on the viability of KMRC-1 sublines stably expressing control shRNA (KMRC-1-shRNA) or SGLT2-targeting shRNA (KMRC-1-shSGLT2). SGLT2 knockdown considerably reduced cell viability at 48 h and 72 h compared with the control, as we described earlier [20] (Figure 4). Two-way ANOVA showed significant main effects of both SGLT2 knockdown and STF-31 treatment on cell viability at 48 h (*p* = 0.002 and *p* < 0.001, respectively) and 72 h (*p* = 0.004 and *p* < 0.001, respectively). In contrast, no significant interaction between SGLT2 knockdown and STF-31 treatment was observed at 48 or 72 h (*p* = 0.520 and *p* = 0.879, respectively). At 24 h, neither main effect nor their interaction was statistically significant.

### 2.6. SLC5A2 Expression in RCC Tissues

**Figure 5:**
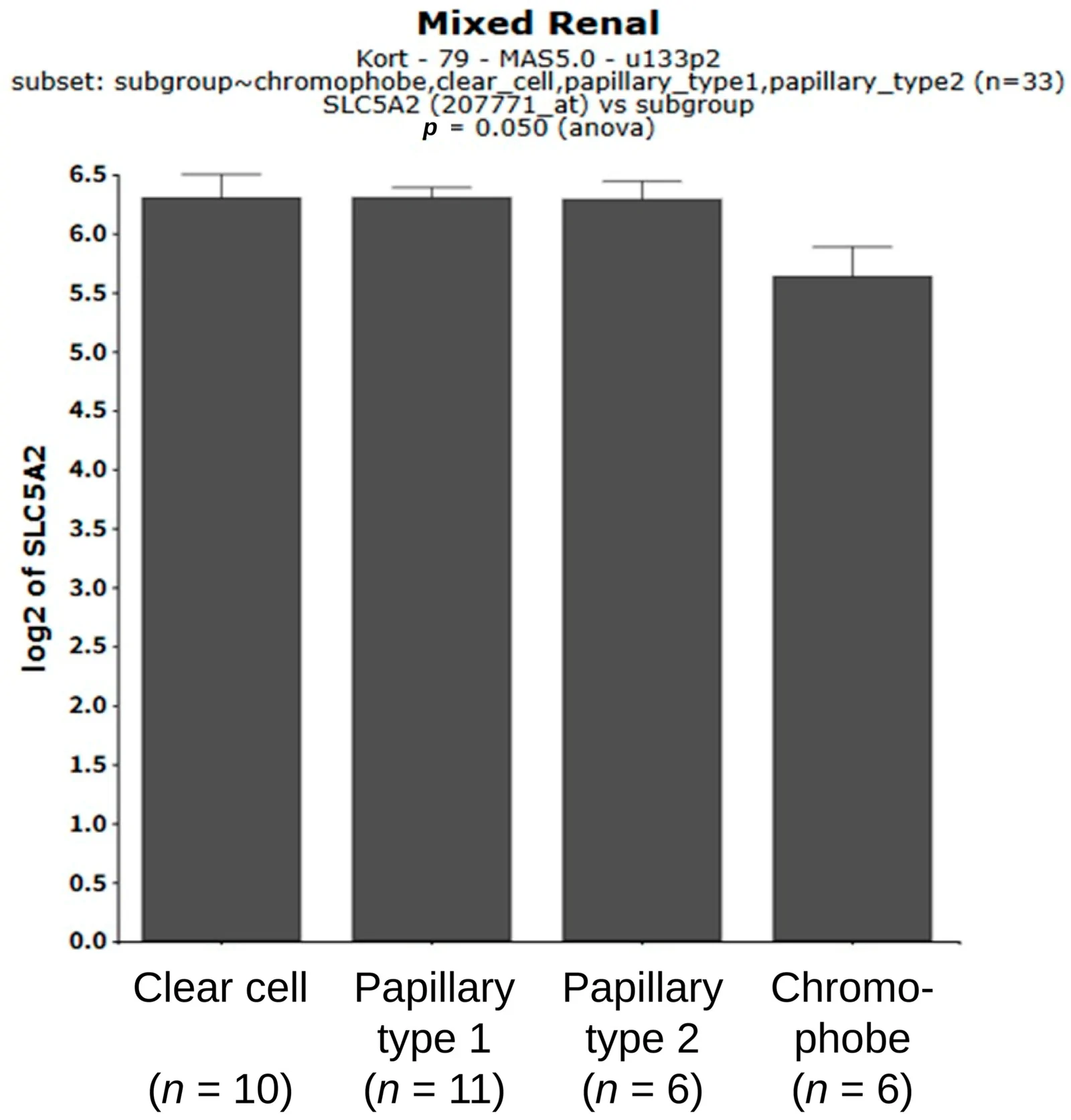
*SLC5A2* mRNA expression across RCC subtypes. *SLC5A2* expression in clear cell, papillary type 1, papillary type 2, and chromophobe RCC in the GSE11024 dataset. Data were analyzed using the R2 Genomics Analysis and Visualization Platform.

A publicly available database was used to examine *SLC5A2* expression in ccRCC and other RCC subtypes. Analysis of the GSE11024 dataset [24] demonstrated detectable *SLC5A2* mRNA expression across RCC subtypes (Figure 5). Although chromophobe RCC showed a trend toward lower *SLC5A2* expression, no statistically significant differences were observed among RCC subtypes after correction for multiple comparisons (Table 2). Notably, chromophobe RCC originates from intercalated cells of the kidney [4,25], whereas ccRCC and most papillary RCC arise from PTECs [4,26].

## 3. Discussion

This study showed that the glycogen-rich clear cell morphology of ccRCC is associated with both loss of functional VHL and SGLT2 expression. In VHL-deficient KMRC-1 cells, SGLT2 inhibition or knockdown and VHL restoration reduced glycogen-associated PAS staining and cytoplasmic area. Paired intratumoral analysis of human ccRCC specimens further showed higher SGLT2 immunohistochemical scores in clear cell than in adjacent eosinophilic components, whereas GLUT1 scores did not differ significantly. Together, these findings suggest that SGLT2-mediated glucose uptake contributes to glycogen accumulation within the VHL-deficient metabolic context of ccRCC.

ccRCC exhibits considerable clinical heterogeneity in terms of both disease progression and treatment response, which corresponds to marked molecular variability at both the intertumoral and intratumoral levels [27,28,29]. Remarkably, ccRCC has a distinct mutational landscape; genes that are frequently mutated in other cancers are rarely affected in ccRCC, whereas mutations common in ccRCC, such as those involving *VHL*, are seldom found in other tumor types [30]. Similarly, the glycogen-associated clear cell phenotype is not a typical histological feature of malignancies arising in other organs, even in patients with VHL disease. Although this morphology is attributed to the cytoplasmic accumulation of glycogen and lipids, the underlying mechanisms remain unclear. We have provided the first evidence of a relationship between SGLT2 expression and ccRCC morphology, with a particular emphasis on the cytoplasmic accumulation of glycogen.

Although the mechanisms and significance of the cytoplasmic accumulation of glycogen in ccRCC have not been fully elucidated, several researchers have proposed potential explanations and implications. For instance, an in vitro study demonstrated that RCC cell lines with *VHL* mutations contained significantly higher levels of cytoplasmic glycogen than those with wild-type *VHL* [15]. Notably, these *VHL*-mutated ccRCC cells demonstrated resistance to glucose deprivation conditions [15]. Hence, glycolysis in ccRCC cells with *VHL* mutations might be supported by larger carbon stores under hypoglycemic conditions. Conversely, unlike other cancer types, such as glioblastoma and non-small cell lung cancer, disrupting the synthesis or breakdown pathways of glycogen appeared to exert limited or no effect on the growth of ccRCC cells, which suggests that glycogen accumulation is a bystander effect of other metabolic changes driven by HIF activity in ccRCC [22]. Although the authors of these studies investigated the association between *VHL* mutations and cytoplasmic glycogen accumulation in ccRCC, they did not explore the impact of SGLT2 on glycogen accumulation. Furthermore, although glycogen accumulation may be a bystander phenomenon, it is driven by excessive glucose uptake, which is essential for cell survival. Therefore, it is crucial to understand the mechanisms and biological significance of glucose uptake. One proposed mechanism involves the upregulation of GLUT1 through the activation of HIF [13]. Nevertheless, GLUT1 is overexpressed in various cancers [31,32,33]. If glycogen accumulation were merely caused by increased GLUT1 expression and metabolic reprogramming due to the loss of VHL function, a similar accumulation would be observed in tumors outside the kidney in patients with VHL disease. Hence, although glycogen accumulation itself might not be functionally significant, its underlying mechanisms may provide important insights into the pathophysiology of ccRCC. On the basis of our findings, one such mechanism may involve SGLT2, a highly active glucose transporter with expression limited to specific tissues and tumor types, which likely contributes to increased glucose uptake and cytoplasmic glycogen accumulation in this type of cancer. Importantly, our previous study showed that SGLT2 inhibition suppressed ccRCC proliferation and migration in vitro, providing a rationale for further investigating SGLT2 as a potential therapeutic target in ccRCC [20]. Unlike GLUT1 inhibitors, whose clinical translation has been limited by the ubiquitous expression of GLUT1 in normal tissues because of concerns regarding systemic toxicity and a narrow therapeutic window [34], SGLT2 inhibitors are already widely used in clinical practice for the treatment of diabetes mellitus, chronic kidney disease, and heart failure, with well-established efficacy and safety profiles [35]. These observations support further investigation of the potential therapeutic relevance of SGLT2 inhibition in ccRCC. However, the present study does not establish antitumor efficacy in vivo or identify predictive biomarkers of treatment response. Further validation in additional preclinical models and prospective clinical studies will be required.

Reprogramming of cellular energy metabolism, including glucose metabolism, has been recognized as a hallmark of cancer [36]. Even in the presence of oxygen, cancer cells predominantly generate energy through glycolysis—a phenomenon known as the “Warburg effect” or aerobic glycolysis [37]. To produce substantial amounts of adenosine triphosphate (ATP) via glycolysis, cancer cells upregulate glucose transporters, particularly GLUT1, to increase glucose uptake into the cytoplasm. ccRCC is considered a prototypical malignancy that exhibits such metabolic reprogramming. As mentioned earlier, most ccRCC tumors harbor loss of VHL function, resulting in the aberrant accumulation of HIF-1α and HIF-2α [10,11,12]. Consistent with this phenomenon, ccRCC exhibits increased levels of glycolytic metabolites and enzymes, as well as increased expression of GLUT1 [13]. ccRCC upregulates the expression of GLUT1, hexokinase 1, and hexokinase 2 but suppresses that of glucose-6-phosphatase, thereby increasing the production of glucose-6-phosphate, a precursor for glycogen. These enzymatic changes enable ccRCC to maintain glucose uptake beyond the levels essential for cellular survival. Furthermore, ccRCC increases glycogenesis through the overexpression of glycogen synthase 1, which has been demonstrated to promote tumor growth [21].

Glucose uptake is essential for cellular metabolism and is mediated by two major families of glucose transporters: facilitative glucose transporters (GLUTs) and sodium–glucose cotransporters (SGLTs) [38]. In humans, 14 GLUT isoforms exist as membrane transporters that facilitate glucose transport along a concentration gradient. In contrast, three SGLTs have been identified that which actively transport glucose against its chemical gradient by coupling glucose uptake to the sodium electrochemical gradient generated by the sodium–potassium ATPase. GLUT1 is ubiquitously expressed in most tissues and overexpressed in various cancers, including ccRCC [31,32,33]. GLUT1 mediates basal glucose uptake into several tissues to maintain glucose homeostasis [39]. SGLT2 is a low-affinity, high-capacity glucose cotransporter that mediates glucose uptake indirectly coupled to ATP consumption. Remarkably, the Michaelis constants (Km) for glucose differ among glucose transporters, with the Km values in human GLUT1 and SGLT2 for D-glucose being 1–2 mM (18–36 mg/dL) and 5 mM (90 mg/dL), respectively [38,40,41,42]. As the Km value of GLUT1 is less than the normal blood glucose concentration, the high-affinity transporter functions at a rate close to maximal velocity. Interestingly, the GLUT1 inhibitor STF-31 has been reported to exert marked cytotoxic effects against *VHL*-mutated ccRCC cells [43]. In the present study, both SGLT2 knockdown and STF-31 treatment showed significant main effects on cell viability at 48 and 72 h. However, no significant interaction between SGLT2 knockdown and STF-31 treatment was observed at any time point (Figure 4). Thus, the present viability assay does not provide evidence for a functional interaction between SGLT2 and GLUT1. The absence of a statistical interaction does not establish that the two transporters function through completely independent pathways or exclude an upstream–downstream relationship. Further mechanistic studies will be required to clarify their relationship. Kobayashi et al. reported that all ccRCC tissues were positive for immunohistochemical GLUT1 expression [19]. Consistent with this report, our immunohistochemical findings demonstrated that all the clear cell components expressed GLUT1, as well as SGLT2 (Figure 2). Taken together, these findings suggest that both GLUT1 and SGLT2 contribute to glucose uptake in ccRCC, although their functional relationship remains unclear. Our exploratory analysis of the GSE11024 dataset showed that *SLC5A2* is expressed not only in ccRCC but also in other RCC subtypes, with no statistically significant differences after correction for multiple comparisons (Figure 5, Table 2). Although these findings should be interpreted cautiously, the presence of *SLC5A2* expression across multiple RCC subtypes suggests that SGLT2 expression alone is insufficient to account for the characteristic glycogen-rich clear cell morphology of ccRCC. SGLT2 expression may reflect, at least in part, the proximal tubular lineage of renal tumors, whereas the characteristic VHL loss in ccRCC provides a distinct metabolic context that may cooperate with SGLT2-mediated glucose uptake to promote glycogen accumulation. SGLT2-mediated glucose uptake may therefore cooperate with VHL-associated metabolic reprogramming to promote glycogen accumulation and contribute to the glycogen-rich clear cell morphology of ccRCC. The present data do not establish a direct upstream–downstream relationship between VHL and SGLT2 or demonstrate that these factors act through completely independent pathways. Because VHL-deficient and VHL-proficient renal cancer cells were not directly compared in this study, differences in SGLT2 expression or function according to VHL status remain unclear. Further studies examining SGLT2, HIF-1α/HIF-2α, and GLUT1 following VHL manipulation will be required to clarify this relationship.

The strengths of this study include the integration of in vitro and ex vivo evaluations, which demonstrated a consistent association between ccRCC morphology and SGLT2 expression. Furthermore, our in vitro findings support a role for SGLT2 in glucose uptake in KMRC-1 cells. Moreover, for the evaluation of clinical specimens, we aimed to minimize background variability by selecting regions within primary tumors that demonstrated morphological intratumoral heterogeneity. This approach enabled the investigation of target molecules such as SGLT2 under conditions in which genetic alterations, including those in *VHL*, were considered closely matched as possible for comparisons.

This study also has several limitations. First, the in vitro analyses were performed using a single VHL-deficient ccRCC cell line, KMRC-1. KMRC-1 exhibits characteristic clear cell morphology with abundant cytoplasmic glycogen accumulation, as previously reported [15], making it a suitable model for investigating the VHL-deficient, glycogen-rich clear cell phenotype of ccRCC. Nevertheless, ccRCC is genetically and metabolically heterogeneous, and cell-line-specific effects cannot be excluded. Future validation using additional VHL-deficient ccRCC cell lines with diverse genetic backgrounds will strengthen the generalizability of our findings. Second, glucose uptake was not directly assessed in SGLT2-knockdown cells. In addition, fluorescence intensity in the glucose uptake assay was not normalized to cell number or protein content; the 96 h dapagliflozin treatment may have affected cell number, which may have influenced the measured fluorescence signal. Third, the number of clinical specimens was limited, although intratumoral comparisons were specifically designed to minimize intertumoral heterogeneity. Furthermore, because the clear cell components were low grade, whereas the adjacent eosinophilic components were high grade, it is not possible to distinguish whether the lower SGLT2 expression in eosinophilic components observed in this study is associated specifically with morphological change or with higher tumor grade/dedifferentiation. Validation in larger independent cohorts will be necessary to confirm the reproducibility and clinical applicability of these findings. In addition, the characteristic clear cytoplasm of ccRCC reflects the accumulation of both glycogen and lipids, whereas the present study focused specifically on glycogen accumulation. Because lipid accumulation and lipid metabolism were not evaluated, our findings address the glycogen-associated component of the clear cell morphology rather than the complete clear cell phenotype. Finally, in vivo validation using animal models was not performed. Future studies incorporating multiple ccRCC cell lines, larger clinical cohorts, and in vivo models will further clarify the role of SGLT2 in glycogen accumulation and glycogen-rich clear cell morphology in ccRCC.

In summary, our findings suggest that SGLT2-mediated glucose uptake contributes to glycogen accumulation and the glycogen-rich clear cell morphology of VHL-deficient ccRCC. These data provide insights into the metabolic basis of the characteristic morphology of ccRCC. The potential therapeutic relevance of SGLT2 inhibition in ccRCC remains to be determined and will require further validation in additional preclinical models and prospective clinical studies.

## 4. Materials and Methods

### 4.1. Cell Cultures and Chemicals

KMRC-1 is a human ccRCC line with mutant *VHL* [15], and ACHN is a human RCC line with wild-type *VHL* [23]. KMRC-1 and ACHN cell lines were purchased from the Japanese Collection of Research Bioresources Cell Bank (Ibaraki, Osaka, Japan) and the American Type Culture Collection (Manassas, VA, USA), respectively. Both cell lines were authenticated by short tandem repeat profiling by the respective cell banks prior to purchase and were routinely tested for mycoplasma contamination. In KMRC-1, a wild-type human *VHL* (sc-400528-LAC, Santa Cruz Biotechnology, Dallas, TX, USA) (i.e., KMRC-1-VHL), or a control lentiviral activating particle (sc-437282, Santa Cruz Biotechnology) (i.e., KMRC-1-control), was stably expressed, as described in our previous study [44]. Successfully transduced cells were selected with puromycin. Sublines stably expressing SGLT2-short hairpin RNA (shRNA) (i.e., KMRC-1-SGLT2-shRNA) or non-silencing control-shRNA (i.e., KMRC-1-control-shRNA) were established in our previous study [20]. All experiments were conducted within 20 passages. KMRC-1 and its sublines and ACHN were cultured in Dulbecco’s modified Eagle’s medium (DMEM; FUJIFILM Wako Pure Chemical Corporation, Osaka, Japan) supplemented with 10% heat-inactivated fetal bovine serum and penicillin (100 units/mL)–streptomycin (100 μg/mL). Cells were maintained at 37 °C in an incubator in a humidified atmosphere of 5% CO_2_. The SGLT2 inhibitor dapagliflozin and the GLUT1 inhibitor STF-31 were obtained from ChemScene (Monmouth Junction, NJ, USA) and MedChemExpress (Monmouth Junction, NJ, USA), respectively.

### 4.2. Western Blotting

Proteins were extracted using RIPA buffer containing a protease and phosphatase inhibitor cocktail (Halt Protease and Phosphatase Inhibitor cocktail; Thermo Fisher Scientific, Waltham, MA, USA). The Simple Western™ system was used to detect protein signals [45]. Signals were visualized using a Wes system with the Compass software (version 6.1.0) (ProteinSimple, San Jose, CA, USA). The primary antibodies to VHL (clone VHL40, dilution 1:50, Santa Cruz Biotechnology) and GAPDH (clone G-9, dilution 1:1000, Santa Cruz Biotechnology) were used.

### 4.3. Periodic Acid–Schiff with Diastase Staining

Cells were seeded in an 8-well Nunc™ Lab-Tek™ Chamber Slides System (Thermo Fisher Scientific). After 24 h of culture, the cells were treated with 2 μM of dapagliflozin for 96 h and fixed with 100% ethanol for 30 min at room temperature before PAS-D staining. For PAS-D staining, the fixed cells were incubated with diastase solution for 15 min at 37 °C, washed three times in phosphate-buffered saline, rinsed in deionized (DI) water, and then subjected to PAS staining. Next, the cells were exposed to 1% periodic acid for 15 min at room temperature, followed by washing under running tap water for 5 min and a quick rinse in deionized water. The cells were then stained with Schiff’s reagent for 45 min at room temperature, followed by washing under running tap water for 5 min at 40 °C. Hematoxylin counterstain was added and left for 10 min. The cells were then washed with tap water, dehydrated in alcohol, cleared in xylene, and mounted. Quantitative image analysis of PAS-stained cells was performed using Fiji (ImageJ version 1.54p; National Institutes of Health, Bethesda, MD, USA). Cytoplasmic areas were defined by excluding nuclear regions from whole-cell regions of interest (ROIs). PAS staining intensity within the cytoplasmic ROIs was quantified after color deconvolution. For each condition, three non-overlapping fields were evaluated, and five cells were analyzed in each field, yielding 15 cells per condition per experiment. Cytoplasmic area and mean cytoplasmic PAS staining intensity were quantified separately. Measurements from the 15 cells were averaged within each experiment, and the experiment-level means were used as biological replicates (*n* = 3). For graphical presentation, PAS staining intensity was normalized to the corresponding mock control within each experiment and expressed as a percentage. Statistical analyses of PAS staining intensity were performed using the raw intensity values.

### 4.4. Glucose Uptake Assay

Cells were seeded onto 35 mm uncoated glass-bottom culture dishes (Matsunami Glass Industry, Kishiwada, Osaka, Japan) and cultured for 24 h, after which they were treated with 2 μM dapagliflozin or mock for 96 h, washed twice with serum- and glucose-free DMEM (Wako), and incubated for 15 min in the same DMEM. Next, the cells were treated with the Glucose Uptake Assay Kit–Green (Dojindo Laboratories, Kumamoto, Japan) diluted 1:500 with the same DMEM for 15 min, washed with WI solution (Dojindo Laboratories), and observed by fluorescence microscopy. Intracellular fluorescence was visualized using a BZ-X800 all-in-one microscope (KEYENCE Corporation, Osaka, Japan). For the quantitative evaluation of glucose uptake, the cells were seeded in optical-bottom 96-well plates (cat. 165305, Thermo Fisher Scientific) and treated with the Glucose Uptake Assay Kit–Green in the same above-described manner. Fluorescence intensity (excitation, 488 nm; emission, 520 nm) was measured using a microplate reader (Varioskan LUX, VLBL00D1, Thermo Fisher Scientific). Fluorescence intensity was not normalized to cell number or protein content. The assay was performed in three independent experiments.

### 4.5. Morphological and Architectural Evaluation and Immunohistochemical Analysis of ccRCC Surgical Specimens

We reviewed primary tumor specimens from 29 patients who underwent radical nephrectomy for sporadic ccRCC at the University of Occupational and Environmental Health between January 2023 and December 2024. Among these 29 cases, 6 demonstrated morphologically heterogeneous ccRCC with adjacent clear cell and eosinophilic components and were included in the present analysis. None of these six patients had received any treatment for ccRCC or had been prescribed SGLT2 inhibitors before surgery. This study was approved by the Institutional Review Board of University of Occupational and Environmental Health (UOEHCRB21-074). All six patients were histologically diagnosed with ccRCC. Each renal tumor was graded according to the World Health Organization/International Society of Urological Pathology (WHO/ISUP) grading criteria [46]. Grades I and II are considered low-grade tumors with good prognosis, while Grades III and IV are considered high-grade tumors with poor prognosis [47]. Immunohistochemistry was performed as described previously [48]. Briefly, 4 μm thick formalin-fixed paraffin-embedded tissue sections were stained using primary antibodies to SGLT2 (clone D-6, dilution 1:50; Santa Cruz Biotechnology), GLUT1 (clone A-4, dilution 1:50, Santa Cruz Biotechnology), or VHL (clone Ig32, dilution 1:25; BD Biosciences, San Jose, CA, USA), followed by secondary antibody–peroxidase-linked polymers. Appropriate positive controls for the stains (e.g., PTECs for glucose transporters and VHL) [18,49,50] and negative tissue elements (e.g., adjacent renal stroma and interstitium for glucose transporters and VHL, respectively) [18,50,51] were evaluated and found to be adequate for interpretation in ccRCC. The immunohistochemical staining intensity of SGLT2 and GLUT1 was semiquantitatively scored as follows: 0, negative; 1+, weak; 2+, moderate; 3+, strong. Clear cell and eosinophilic components were separately evaluated within each tumor. All stains were manually evaluated by an experienced and board-certified pathologist (A.N.) who was blinded to sample identification.

### 4.6. Cell Viability

Cell viability was evaluated using the 3-(4,5-dimethylthiazol-2-yl)-2,5-diphenyltetrazolium bromide (MTT; Sigma-Aldrich, St. Louis, MO, USA) assay. Cells (3–5 × 10^3^ cells/well) were seeded in 96-well tissue culture plates and incubated for 24 h, 48 h, and 72 h, followed by the addition of 10 μL MTT of stock solution (5 mg/mL) to each well and incubation for 3 h at 37 °C. The medium was replaced with 100 μL of dimethyl sulfoxide and incubated for 5 min at room temperature. Absorbance was measured at a wavelength of 570 nm, with background subtraction at 630 nm. Each experiment was independently performed three times, with triplicate wells per condition.

### 4.7. Public Database Analysis

The R2 Genomics Analysis and Visualization Platform (https://hgserver1.amc.nl/cgi-bin/r2/main.cgi, accessed on 8 July 2026) was used to determine *SLC5A2* expression in RCC. The dataset was obtained from the Gene Expression Omnibus (GEO) repository (accession no. GSE11024 [24]).

### 4.8. Statistical Analysis

Differences in continuous variables between two groups were evaluated using Student’s *t*-test. Differences in immunohistochemical scores between paired clear cell and eosinophilic components were evaluated using the Wilcoxon signed-rank test. Cytoplasmic area and cytoplasmic PAS staining intensity were analyzed using one-way repeated-measures ANOVA followed by Bonferroni-adjusted pairwise comparisons. The effects of SGLT2 knockdown and STF-31 treatment on cell viability were analyzed separately at each time point using two-way ANOVA, with SGLT2 knockdown and STF-31 treatment as the two factors, including their interaction. Differences in *SLC5A2* mRNA expression among RCC subtypes in the GSE11024 dataset were assessed by one-way ANOVA within the R2 Genomics Analysis and Visualization Platform. Pairwise comparisons were performed within the R2 platform using Welch’s test with Bonferroni correction for multiple comparisons. All other statistical analyses were performed using EZR version 1.54 (Jichi Medical University, Tochigi, Japan). *p* < 0.05 was considered statistically significant.

## Figures and Tables

**Table 1:** Clinicopathological characteristics of patients with heterogeneous clear cell renal cell carcinoma. Tissue samples were obtained from six patients with morphologically heterogeneous ccRCC who underwent radical nephrectomy at the University of Occupational and Environmental Health between 2023 and 2024. Tumors were graded according to the WHO/ISUP grading system.

Patient	Age (Years)	Sex	Clinical Presentation	Stage	WHO/ISUP Grade	
					Clear Cytoplasmic	Eosinophilic
1	53	Female	Weight loss	pT3aN0M0	2	3
2	61	Female	Incidental detection	pT1aN0M0	2	3
3	62	Female	Gross hematuria	pT3aN0M1	2	4
4	70	Female	Palpable mass	pT3aN0M0	2	4
5	77	Female	Weight loss	pT3aN0M0	2	4
6	80	Male	Gross hematuria	pT3aN0M0	2	4

**Table 2:** Statistical analysis of *SLC5A2* expression among RCC subtypes. Differences in *SLC5A2* mRNA expression among four RCC subtypes in GSE11024 were assessed by one-way ANOVA. Pairwise comparisons were performed using Welch’s test with Bonferroni correction for multiple comparisons. Abbreviations: RCC, renal cell carcinoma; NA, not applicable.

ANOVA				
Source of Variation	Sum of Squares	df	Mean Square	F Value
Between groups	2.166	3	0.722	2.927
Within groups	7.155	29	0.247	NA
Pairwise comparisons				
Comparison	Unadjusted *p* value (Welch)		Bonferroni-adjusted *p* value	
Clear cell vs. Papillary type 1	0.990		1.000	
Clear cell vs. Papillary type 2	0.962		1.000	
Clear cell vs. Chromophobe	0.064		0.384	
Papillary type 1 vs. Papillary type 2	0.934		1.000	
Chromophobe vs. Papillary type 1	0.045		0.269	
Chromophobe vs. Papillary type 2	0.057		0.344	

## Data Availability

The publicly available data supporting the findings of this study are available through the R2 Genomics Analysis and Visualization Platform (https://hgserver1.amc.nl/cgi-bin/r2/main.cgi, accessed on 8 July 2026), under GEO accession number GSE11024. The original data generated in the study are available on reasonable request from the corresponding author.
